# Effect of pre- and post-weaning dietary supplementation with arginine and glutamine on rabbit performance and intestinal health

**DOI:** 10.1186/s12917-019-1945-2

**Published:** 2019-06-13

**Authors:** Rebeca Delgado, Rodrigo Abad-Guamán, Nuria Nicodemus, Araceli Diaz-Perales, Javier García, Rosa Carabaño, David Menoyo

**Affiliations:** 1 Departamento de Producción Agraria, ETSI Agronómica, Alimentaria y de Biosistemas, Universidad Politécnica de Madrid, Ciudad Universitaria, Madrid, Spain; 2 Departamento de Biotecnología, Centro de Biotecnología y Genómica de Plantas, Campus de Montegancedo, Universidad Politécnica de Madrid, Pozuelo de Alarcón, Madrid, Spain

**Keywords:** Arginine, Glutamine, Rabbit, Gut health

## Abstract

**Background:**

The purpose of the present study was to assess if the exposure to glutamine (Gln), arginine (Arg) or their combination from pregnancy, through the maternal diet, to a post weaning supplemented diet, can stimulate litter performance, gut development and immune function. To this end does and their litters were fed the same basal diet no supplemented (control C), or supplemented with 0.4% Gln, 0.4% Arg, or 0.4 Gln + 0.4 Arg. Rabbits were weaned at 25 d of age and fed the same experimental diet as their mothers for 10 additional days (35 d of age). Bacterial translocation to mesenteric lymph nodes (MLN) at 6 d of age and intestinal histology, enzymatic activity, phenotypical and functional analysis of intraepithelial lymphocytes (IEL) from the appendix were determined at 6, 25 and 35 d of age.

**Results:**

No significant differences on animal performance or mortality rates were observed among dietary treatments. However, kits from rabbit does supplemented with Gln tended (*P* ≤ 0.10) to reduce the translocation of total number of both aerobic and facultative anaerobic bacteria to the MLN. Also, rabbits fed the Gln supplemented diets maintained intestinal villous height at weaning compared to the non-supplemented diets (*P* < 0.05). The proportions of CD45^+^CD4^+^ and CD45^+^CD8^+^ IEL in the appendix were not affected by dietary means. However, in rabbits IEL at weaning dietary Gln significantly upregulated IL-2 and downregulated IL-6 expression.

**Conclusions:**

Despite a lack of effect on performance and mortality the inclusion of 0.4% Gln has a positive effect by maintaining intestinal villous height and modulating the cytokine profile at weaning. The supplementation with Arg or Arg + Gln at the selected doses in this study did not exert positive effects on rabbit intestinal health.

## Background

The use of feed additives is becoming a common practice to prevent diseases in young productive animals [1]. Amino acids with functional properties such as arginine (Arg) and glutamine (Gln) have been proven to be nutritionally essentials for neonates and under stressful conditions such as weaning [2–5].

Despite Arg can be synthetized from other amino acids (Gln, glutamate and proline) it is considered an essential amino acid in young animals as their needs exceeds endogenous synthesis [3, 6]. Arginine is an important N carrier and precursor for amino acids, proteins and polyamines needed for immune cell proliferation [2]. Also, it is a substrate for the synthesis of nitrogen oxide (NO) a powerful mediator in both innate and adaptive immunity [6]. Arginine supplementation (0,5–1% w/w) to young pigs supports intestinal epithelial growth and maintains gut barrier integrity and function against bacterial toxins [6].

Glutamine is also considered an indispensable amino acid for intestinal development and function [2, 6, 7]. It provides energy and it is the precursor for other amino acids (including citrulline, Arg and glutamate) and derivatives needed for enterocyte and immune cells function and proliferation [2, 6]. Moreover, it has been proven to play important roles under disease and stress situations by maintaining epithelial integrity and gut associated lymphoid tissue (GALT) function [3, 6, 7]. Recently, beneficial effects of Gln on gut health have also been associated to a direct use of this amino acid by commensal bacteria [8].

Several authors observed that the combination of Arg and Gln have beneficial additive effects [9–11]. Our previous research with growing rabbits showed that diets supplemented with 1% of Gln and 0.5% of Arg tend to improve productive performances reducing the presence of *Clostridium spp*. and *Helicobacter ssp*. in the caecum and in the ileum [12]. Moreover, ex vivo experiments suggest that the combination of Arg and Gln decrease the production of pro-inflammatory cytokines [13].

The aim of this study was to determine if the rabbit gastrointestinal tract development and function is positively affected by feeding pre-weaning (pregnancy period) and post-weaning diets supplemented with Arg, Gln and their interaction.

## Methods

### Animals and housing

Rabbits were handled following the principles and guidelines of care of animals in experimentation (Spanish Royal Decree, 53/2013, BOE, 2013) which meets the European Union Directive 2010/63/EU about the protection of animals used in experimentation. Rabbits (New Zealand White × Californian, V × R genetic hybrids from Universidad Politécnica de Valencia, Valencia, Spain) were offspring of primiparous does breed at our facilities. They were nursed by their mothers until 25 days of age. Rabbit does were housed individually and kept under controlled environmental conditions with 18–23 °C and 16 h daily lighting. An external nestbox with wood shavings was provided three days before parturition. Nulliparous rabbit does were assigned randomly to the four treatments (20 per diet) at 123 d of age, 6 days before the first artificial insemination (AI), with a body weight (BW) of 3.6 ± 0.1 kg. Forty eight h before insemination, the does were injected 25 IU of equine chorionic gonadotropin (Segiran, Lab. Ovejero, León) to synchronize oestrus. The day of insemination, does received an intramuscular injection of 1 μg of buserelin Suprefact® (Hoechst Marison Roussel, S.A., Madrid), a Gonadotropin-releasing hormone agonist (GnRH agonist), to induce ovulation in rabbit does [14]. At the time of weaning (25 days of age) rabbits were housed collectively in groups of 2 or 3 animals per cage (650 mm × 250 mm × 330 mm). Four hundred and seventy one rabbits weighting 390 ± 82 g were allocated into 190 cages and assigned the same dietary treatment as during the lactating (pre-weaning) period. Housing conditions were controlled during the whole experimental period. The farm temperature was maintained between 18 and 24 °C with 12 h of light and 12 h of darkness.

Therefore, in total 559 rabbits were used in this study, of them 88 were slaughtered at 6 and 25 days of age to collect samples. The remaining 471 were used to analyze growth performance (from 25 to 35 days of age). A final sampling of 40 rabbits with 35 days of age was done to complete the 6, 25 and 35 days of age sample set to analyze dietary effects on selected intestinal health variables. The maximum number of replicates was fixed for weight gain assuming changes in means expected between groups of 3.5 g per day and a standard deviation of 6 using a power of test of 80% (1-beta) and a significant level of 0.05 (alpha).

### Experimental diets and growth performance

Four diets were used following a 2 × 2 factorial arrangement (two levels of Arg combined with two levels of Gln). A control diet (C) was formulated to contain 19.1% protein and 35.3% NDF (on DM basis) and meeting the minimal nutrient requirements [15]. Three other diets were obtained by adding on top to the control diet 0.4% Arg (Arg diet), 0.4% Gln (Gln diet) or 0.4 Gln + 0.4 Arg (Gln + Arg diet). L-Glutamine and Arg were provided by Indukern S.A. (Barcelona, Spain). Arginine and Gln doses were fixed according to the positive effects on rabbit health observed in previous studies [14]. The ingredient and chemical composition of experimental diets are shown in Tables 1 and 2. Rabbits had ad libitum access to feed and water, and each litter received the same diet as its mother. Therefore, rabbits were exposed to Arg and Gln or their combination during lactation through the maternal diet and after weaning. Feed intake, body weight gain and feed efficiency were estimated 10 d after weaning (day 35), whereas mortality was recorded daily.

**Table 1:** Ingredient composition of the control diet (g/kg DM)

Ingredients	C
Alfalfa hay	290
Wheat	220
Wheat straw	220
Sunflower meal 28–30	130
Soybean meal 44	65.0
Soy protein concentrate 61	15.0
Defatted grape seed meal	20.0
Lard	20.0
L-Lysine HCl	2.00
DL-Methionine	0.50
L-Threonine	0.50
Calcium carbonate	6.80
Sodium chloride	5.00
Vitamin/mineral premix^a^	5.00
Coccidiostat^b^	0.20

^a^Provided by Trouw Nutrition-Tecna (Madrid, Spain). Mineral and vitamin composition (mg/kg): Mn: 4000; Zn: 11840; Cu: 2000; I: 250; Co: 99; Fe: 15200; Niacin: 4000; Betaine: 10830; Choline: 27500; Vitamin K: 200; Vitamin B1: 200; Vitamin B2: 400; Vitamin B6: 200; Vitamin A: 1675000UI/kg; Vitamin D3: 150000 UI/kg; Vitamin E (α-tocopherol acetate): 4000UI/kg.^b^ 1 ppm diclazuril provided Esteve (Barcelona, Spain)

**Table 2:** Chemical composition of experimental diets (g/kg DM)

Diets	C	Arg	Gln	Arg + Gln
Arginine	0	0.4	0	0.4
Glutamine	0	0	0.4	0.4
Analyzed composition, g/kg DM				
Dry matter	899	893	897	901
Ash	67.5	71.2	68.9	71.0
Nitrogen	29.8	30.6	30.1	30.4
Ether extract	50.0	50.0	47.0	45.0
Total dietary fibre	418	423	426	425
Neutral detergent fibre	313	322	319	321
Acid detergent fibre	179	174	176	179
Acid detergent lignin	48.1	44.6	45.6	44.1
Soluble fibre	105	101	107	105
Gross energy (MJ/kg DM)	18.0	18.5	18.4	18.4
Amino acids, g/kg DM				
Alanine	8.00	7.90	8.00	8.00
Arginine	11.3	15.3	11.6	15.3
Aspartic acid	16.1	16.0	15.9	16.1
Cystine	2.90	2.90	2.90	2.90
Glutamic acid	30.8	30.3	34.5	35.3
Glycine	8.70	8.60	8.70	8.70
Histidine	4.00	3.90	4.00	3.90
Isoleucine	6.90	6.70	6.80	6.60
Leucine	11.9	11.8	11.8	11.9
Lysine	9.30	9.20	9.20	9.20
Methionine	3.30	3.20	3.20	3.20
Phenylalanine	8.10	8.00	8.10	8.00
Proline	10.4	10.3	10.3	10.4
Serine	7.90	7.80	7.70	8.10
Threonine	7.00	6.90	6.80	7.00
Valine	8.50	8.30	8.40	8.20

### Chemical analysis

Procedures of the AOAC [16] were used to determine the dietary concentrations of DM (934.01), ash (942.05) and CP (954.01). Dietary NDF, ADF and ADL were determined sequentially using the filter bag system (Ankom Technology, New York, NY) [17]. Gross energy was measured by adiabatic bomb calorimeter (model 356, Parr Instrument Company, Moline, IL). Amino acids were determined after acid hydrolysis using a Beckman System 6300HPA AA analyzer (Fullerton, CA). Samples were hydrolyzed by reflux in 25 mL of 6 M HCl with 10 g/L of added phenol for 24 h at 120 °C. For the determination of sulfur AA (Met and Cys), samples were oxidized with per-formic acid at 0 °C for 16 h and then neutralized with 0.5 g of sodium meta-bisulphite before analysis. During acid hydrolysis, tryptophan was destroyed and was not determined.

### Bacterial translocation to mesenteric lymph nodes

Six days after parturition 24 kits, one per litter (24 litters; 6 litters/diet), were randomly selected from out of the 14 litters per treatment included in the study (see [14] for details) and slaughtered by cervical dislocation at 12:00 h a.m. The mesenteric lymph nodes (MLN) located at the base of the small-bowel mesentery was excised completely under sterile conditions for bacterial analysis. The nodes, weighing on average 19 ± 9 mg, were dissected free from fat and placed in a sterile eppendorf containing 1 ml of sterile peptone water. Later, they were homogenized using a mixer mill (Reisch MM 400) by applying a frequency of 22 Hz during 2 min. Each ml of MLN homogenized was blended with 9 ml of peptone water and diluted (3 dilutions per sample). Dilutions were plated onto blood agar (columbia agar + sheep blood, Oxoid S.A.). For aerobes analysis plates were incubated at 37 °C for 48 h in aerobic conditions. For anaerobes analysis plates were incubated in jars of 6 l with anaerobic atmosphere (two AeroGen 3.5 l per jar; Oxoid S.A.). Facultative anaerobes microorganisms were incubated in jars of 6 l with one carbon dioxide generating envelopes (AeroGen 3.5 l; Oxoid S.A.). For each analysis, a blank with sterile peptone water was cultured onto blood agar to verify there was not environmental contamination. After incubation, colonies were counted and recorded as CFU per mg of sample.

### Gut histology and enzymatic activity

Forty-eight rabbits with 6 days of age (12/diet), 40 rabbits with 25 days of age (10/diet) and another 40 rabbits with 35 days of age (10/diet), were randomly selected and killed by cervical dislocation. The remaining rabbits completed the productive cycle and were euthanized by head concussion with approximately 2 kg of body weight. A 3 cm section of middle part of jejunum was collected in 10% buffered neutral formaldehyde solution (pH 7.2 to 7.4) for histological analysis. Also, at 25 and 35 days of age, tissue samples from middle part of jejunum (6 cm each) were collected to determine intestinal sucrose enzymatic activity. This tissue was cleaned with saline solution, snap frozen and stored at − 80 °C.

Collected jejunal samples were gradually dehydrated in an ethanol series (50 to 100%) and infiltrated with paraffin wax using tissue processor LEICA ASP 300. Samples were sectioned at 5 μm with microtome LEICA RM 2255. The slides were stained with Alcian blue to visualize acidic mucins [18], by using an automatic procedure (ArtisanTM Link Special Staining System). The sections were placed in acetic acid-AB2.5, pH 2.5, for 5 min, and then they were placed in alcian blue-AB2.5, pH 2.5 for 10 min, at 37 °C and subsequently washed in water six times. The sections were counterstained with eosin, dehydrated, and covered with a cover slip using nuclear fast red-AB2.5 for 10 min and subsequently washed in water six times [19]. One slide containing jejunal section was prepared for each sample and all of them were viewed at 40X magnification using an Olympus BX-40 light microscope. Images were digitally captured for later analysis using Soft software version 3.2 C4040Z (Soft Imaging System, Olympus, GmbH, Hamburg, Germany), and analyzed eye blinded by the same person. Villous height and crypt depth were measured individually [20] and an average of the measurements was obtained for each animal. The amount of goblet cell from each villi measured were counted. Sucrose enzymatic activity in jejunal samples was analyzed as previously described [21].

### Phenotypical and functional analysis of intraepithelial lymphocytes from the appendix

Appendix was taken from 24 animals (6/diet) at 6, 25 and 35 days of age. After removal, tissue was placed in ice-cold 10 mM PBS, pH 7,4 and immediately processed for intraepithelial lymphocyte isolation. For this, appendix samples were cut in small pieces with a scalpel and incubated in 9% HBSS containing Dispase I (100 u; Sigma, Alcobendas España), DNase I (30μg/ml; Sigma, Alcobendas Spain) and 10% Fetal Calf Serum and HEPES (1,5%; pH = 7.2) [22]. Isolated lymphocytes were count in a Neubauer chamber and stored at − 80 °C in RPMI/ DMSO (12%) at a final concentration of 10^6^ cel/ml.

For the phenotypic characterization of lymphocytes, cells were incubated in commercial monoclonal antibodies and flow cytometry acquisition was performed on an Accuri cytometer (BD Accuri Cytometers, Ann Arbor, MI). The following antibodies were used for flow cytometry: anti-rabbit CD45^+^ (VMRD INC, Pullman WA, USA) for total lymphocytes, CD4^+^ for T helper lymphocytes, and CD8^+^ (AbynteK Biopharma, S.L, Bizkaia, Spain) for cytotoxic T lymphocytes. Data were analyzed by CFlow Plus software, version 1.0.227.4 (BD Bioscience).

For lymphocyte functional response, total RNA was isolated from approximately 2 × 10^6^ cells using the GenElute Mammalian Total RNA Miniprep kit (Sigma-Aldrich, St Louise, MO, USA) according to manufacturer’s instructions. The relative gene expression of selected interleukins (IL) was analyzed by using real-time, quantitative PCR. First strand cDNA was synthesized using the High-Capacity cDNA Archive Kit (Applied Biosystems Foster City, CA, USA) according to the manufacturer’s instructions. Target ILs were selected for their known functional proinflammatory (IL-2, IL-6, IL-8) and antiinflamatory (IL-10) roles in the appendix. The specific primers and reaction conditions for rabbit GAPDH, hypoxanthine phosphoribosyltransferase (HPRT) (housekeeping genes) and IL-10 were obtained from the literature [12, 23]. Those for IL-2, IL-6 and IL-8 were designed by us using Primer Express® v.2 (Applied Biosystems, Foster City, CA, USA) (Table 3). Specific product amplification was checked by the melting curve analysis. The quantitative PCR was performed in an ABI Prism 7300 Sequence Detector System (Applied Biosystems, Foster City, CA, USA). Each reaction mix consisted on around 100 ng of first strand cDNA as a template, specific primers, ultra-purified water and SYBR® Green Master Mix (Applied Biosystems Foster City, CA, USA) as fluorescent DNA intercalating agent. The concentration and annealing temperatures for IL-2 were 0.2 μM and 60 °C; 0.4 μM and 62 °C for IL-6; and 0.2 μM and 60 °C for IL-8. All samples were run in triplicate and quantified by normalizing the cytokine signal of GADPH and HPRT.

**Table 3:** Primers for real-time PCR assay

Gene	GenBank	Forward primer	Reverse primer
HPRT^a^	M31642	5′-TGATAGATCCATTCCTATGACTGTAGA-3′	5′-GGGTCCTTTTCACCAGCAG-3′
GADPH^b^	AB231852	5′-GGG CGT GAA CCA CGA GAA- 3′	5′-GCC GAA GTG GTC GTG GAT-3′
IL-10^a^	D84217	5′-GAGAACCACAGTCCAGCCAT-3′	5′-CATGGCTTTGTAGACGCCTT-3′
IL-6	DQ680161	5′-GAGCATCCTGGAGACCATCAA-3’	5′-CCAGTGCCTCCTTTCTGTTCA-3’
IL-8	Ensembl^c^	5′-GCAACCTTCCTGCTCTCTCTGA-3′	5′-CACTGGCATCGAAGCTCTGTAC-3′
IL-2	Ensembl^d^	5′-CAAACTTTCCAGGATGCTCACA-3’	5′-GAGGTTTGAGTTCTTCTTCTAGACACTGA-3’

^a^Godornes et al., 2007 [23]

^b^Chamorro et al., 2010 [12]

^c^Rabbit Ensembl ENSOCUG0000001 1835

^d^Rabbit Ensembl ENSOCUT00000010098

### Statistical analysis

Rabbit performance (BW, ADG, ADFI and G:F) was analyzed using an analysis of variance (ANOVA) with the GLM procedure of SAS (SAS Inst., Cary, NC) with, Arg level, Gln level and their interactions as a fixed effects. Mortality was analyzed using a logistic model (GENMOD procedure of SAS considering a binomial distribution) including Arg level, Gln level and their interactions in the model. Microbial counts were analyzed using a Poisson model (GENMOD procedure and considering a Poisson distribution). Mucosal morphology and lymphocyte proportions was analyzed using a variance analysis with the GLM procedure of SAS (SAS Inst., Cary, NC) with, Arg level, Gln level, age and their interactions as a fixed effects. Finally, cytokine gene expression was analyzed using an analysis of variance (ANOVA) with the GLM procedure of SAS. The model included Arg level, Gln level, age and their interactions as fixed effects. The differences between treatment means were considered significant at *P* < 0.05. In addition, the Tukey test was used to compare the effects among different ages, or within age when its interaction with any treatment were significant.

## Results

### Finishing performance and mortality

No significant differences on animal performance were observed among dietary treatments (Table 4). Rabbits showed similar weight gain, feed intake, feed efficiency and final body weight during the experimental period averaging 32.4 g, 45.0 g, 0.722 g/g and 739 g respectively (*P* ≥ 0.28). Also, dietary treatment did not affect the mortality from 25 to 35 d of age which was on average 2.33% (*P* ≥ 0.27).

**Table 4:** Effect of arginine and glutamine supplementation on growth performance and mortality from 25 to 35 d of age

	Diets				*P*-value		
	C	Arg	Gln	Arg + Gln	Arg	Gln	Arg × Gln
Arginine	0	0.4	0	0.4			
Glutamine	0	0	0.4	0.4			
N^a^	50	50	44	46			
Body weight (g) 25d^b, c^	386 ± 13	386 ± 12	395 ± 12	393 ± 12	0.96	0.51	0.95
Body weight (g) 35d^b, c^	725 ± 29	745 ± 27	762 ± 27	723 ± 26	0.74	0.79	0.30
Weight gain, g/d^b, c^	31.3 ± 1.2	33.4 ± 1.1	32.7 ± 1.1	32.3 ± 1.1	0.48	0.87	0.30
Feed intake, g/d^b, c^	43.7 ± 1.6	46.6 ± 1.5	45.1 ± 1.5	44.5 ± 1.5	0.48	0.81	0.28
Feed efficiency, g/g^b, c^	0.711 ± 0.01	0.719 ± 0.01	0.732 ± 0.01	0.727 ± 0.01	0.92	0.38	0.69
Mortality, %^d^	3.57 (1.35–9.13)	2.48 (0.80–7.40)	0.90 (0.13–6.11)	2.36 (0.76–7.07)	0.31	0.66	0.27

^a^*N* number of cages (2–3 rabbits/cage)

^b^Values expressed as means ± standard error

^c^N (rabbits/diet): C = 112, Arg = 121; Gln = 111 and Arg + Gln = 127

^d^Mortality rate with 95% confidence interval in brackets

### Bacterial translocation to mesenteric lymph nodes

At 6 d of age bacterial translocation to MLN was observed, with aerobes, anaerobes and facultative anaerobes present on average at 5.73, 5.20, and 7.84 CFU/mg MLN, respectively (Table 5. values expressed as Ln). Kits from rabbit does supplemented with Gln tended to have lower aerobic (2.62 vs 5.74 CFU/mg MLN; *P* = 0.091) and facultative anaerobic bacteria (2.63 vs 5.86 CFU/mg MLN; *P* = 0.10) compared to those fed the no supplemented Gln diets.

**Table 5:** Effect of experimental diets fed to rabbit does on mesenteric lymph nodes (MLN) microbiota of 6-d suckling kits

	Diets				*P*-value		
	C	Arg	Gln	Arg + Gln	Arg	Gln	ArgxGln
Arginine	0	0.4	0	0.4			
Glutamine	0	0	0.4	0.4			
N	6	6	6	6			
Total aerobes^a^	4.47 ± 1.3	7.02 ± 0.3	2.65 ± 3.4	2.59 ± 3.5	0.65	0.09	0.64
Facultative aerobes	4.61 ± 1.4	7.11 ± 0.4	2.82 ± 3.4	2.45 ± 4.1	0.73	0.10	0.63
Total anaerobes	8.65 ± 0.5	7.55 ± 0.8	6.19 ± 1.7	7.63 ± 0.8	0.87	0.22	0.19

^a^Values expressed as Ln (forming colonies unit/mg MLN) ± standard error

### Gut histology and enzymatic activity

**Fig. 1:**
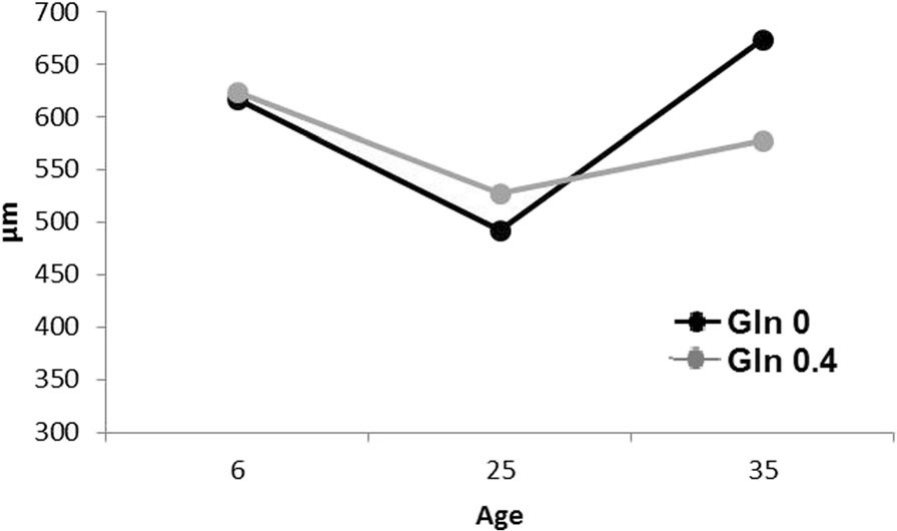
Effect of rabbit age and glutamine supplementation on jejunal villous height. A significant interaction Gln × Age was found (*P* = 0.019) for villous height

Dietary treatments did not affect jejunal villous height, crypt depth or villous height to crypt depth ratio (Table 6). Also, the number of goblet cells and sucrose activity in the jejunum was not affected by diet (Table 6). However, villous height decreased by 17.7%, crypt depth increased 110% and the number of goblet cells per villous increased by 393% (*P* < 0.05) in rabbits at 25 d of age compared to rabbits at 6 d of age. Also, the villous height/crypt depth ratio decreased from 6 to 25 d of age (16.3 vs. 6.36; *P* < 0.05). From 25 to 35 d of age, villous height showed a similar value than observed at 6 d of age and crypt depth increased by 4.7% (*P* < 0.05). This led to a villous height/crypt depth ratio increase from 25 to 35 days of age (6.36 vs. 7.38; *P* < 0.05). The number of goblet cells per villous also increased by 33% from 25 to 35 d of age (*P* > 0.05). Supplementation with Gln maintained villous height at 25 and at 35 days of age compared to the no supplemented Gln diets whose values were lower at 35d of age (*P* = 0.019 for diet x age interaction) (Fig. 1). Sucrose activity increased from 25 to 35 days of age (*P* < 0.001).

**Table 6:** Effects of the experimental diets and age on intestinal morphology and enzyme activity

	Diets				Age			SEM^1^					*P*-value^2^			
	C	Arg	Gln	Arg + Gln	6	25	35	Arg and Gln	Arg × Gln	Age	Arg × Age and Gln × Age	Arg × Gln × Age	Arg	Gln	Arg × Gln	Age
Arginine	0	0.4	0	0.4												
Glutamine	0	0	0.4	0.4												
N	31	32	32	32	48	40	39									
Villous height, μm	609	580	577	575	620^b^	510^a^	626^b^	13.6	19.2	16.6	23.5	33.2	0.44	0.34	0.48	< 0.001
Crypt depth, μm	68.5	67.4	67.8	66.4	38.1^a^	80.2^b^	84.2^c^	0.71	1.01	0.87	1.23	1.74	0.21	0.42	0.86	< 0.001
Ratio villous/crypt	10.2	10.0	9.94	9.96	16.3^c^	6.36^a^	7.38^b^	0.22	0.31	0.27	0.38	0.53	0.76	0.61	0.71	< 0.001
Goblet cells, no/villi	13.7	12.8	12.7	12.7	2.94^a^	14.5^b^	21.6^c^	0.38	0.53	0.46	0.65	0.92	0.41	0.30	0.42	< 0.001
Mucose protein, mg/g of tissue^3^	103	105	102	105	–	102	106	2.80	3.96	2.79	3.95	5.59	0.51	0.86	0.80	0.36
Sucrose activity, μmol of glucose/mg of protein^1^	390	386	314	352	–	116	605	37.8	53.4	37.7	53.4	75.5	0.76	0.31	0.70	< 0.001

^1^*SEM* standard error of the mean

^2^No significant differences (*P* ≥ 0.22) were found for Arg × Age and Arg × Gln × Age interactions. However, a significant (*P* = 0.019) interaction Gln × Age was found for villous height and it is represented in Fig. 1

^3^N = 9 at 25 d of age and *N* = 10 at 35 d of age

^a-c^Diet mean values in the same row with a different superscript differ *P* < 0.05

### Phenotypical and functional analysis of intraepithelial lymphocytes from the appendix

The percentages of CD45^+^CD4^+^ and CD45^+^CD8^+^ intraepithelial lymphocytes (IEL) were not affected by dietary means (*P* = 0.44; Table 7). However, the proportions of CD45^+^CD4^+^ tended to decrease (*P* = 0.065) from 6 to 25 d of age and then to increase again up to 35 d of age. Also, the percentage of CD45^+^CD8^+^ tended to increase (*P* = 0.099) with age.

**Table 7:** Effects of the experimental diets and age on lymphocyte proportions (% of total lymphocytes) in the appendix of rabbits

	Diets				Age			SEM^a^					*P*-value^b^			
	C	Arg	Gln	Arg + Gln	6	25	35	Arg and Gln	Arg × Gln	Age	Arg × Age and Gln × Age	Arg × Gln × Age	Arg	Gln	Arg × Gln	Age
Arginine	0	0.4	0	0.4												
Glutamine	0	0	0.4	0.4												
N	14	14	14	14	20	20	17									
CD45^+^CD4^+^	19.4	20.6	23.7	24.8	23.2	13.6	29.5	3.85	5.44	4.71	6.66	9.41	0.84	0.44	0.99	0.065
CD45^+^CD8^+^	16.1	17.2	20.2	20.7	15.8	12.4	27.5	4.08	5.77	4.99	7.06	9.97	0.89	0.52	0.96	0.099

^a^*SEM* standard error of the mean

^b^No significant differences (*P* ≥ 0.84) were found for Arg × Age, Gln × Age and Arg × Gln × Age interactions

**Fig. 2:**
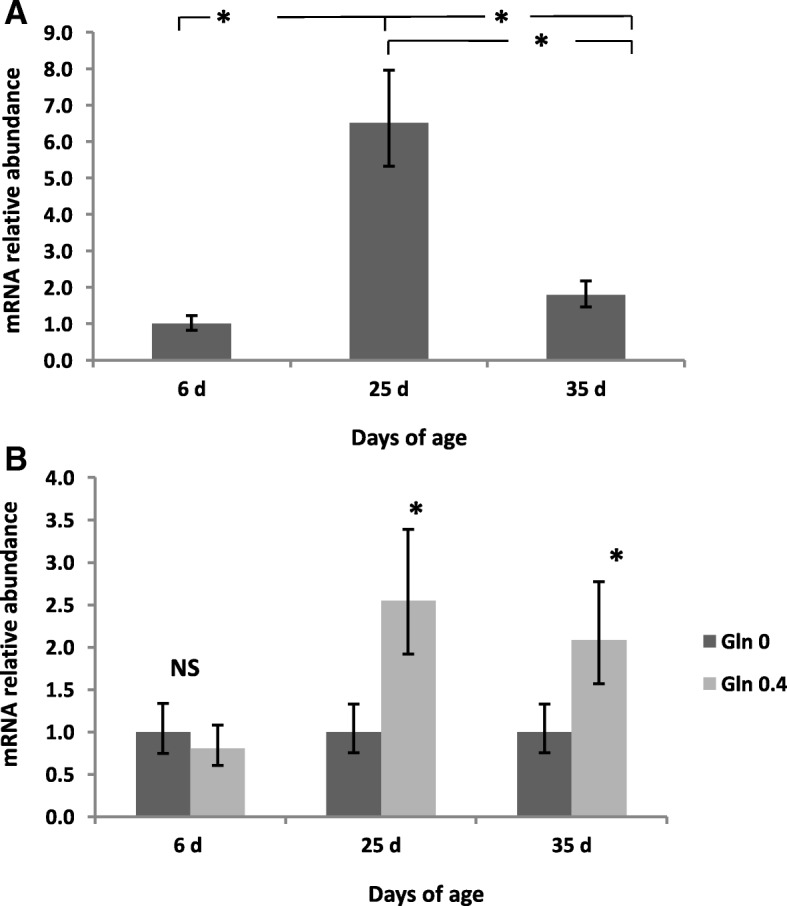
Interleukin (IL)-2 mRNA expression in intraepithelial lymphocytes isolated from rabbit Appendix. A significant effect of age (*P* < 0.001), and of the interaction Gln × Age (*P* = 0.017) were found. **a** Relative gene expression values are fold change of 25 and 35 d relative to 6 d old rabbits set to be 1.0. **b** Relative gene expression values are fold change of rabbits fed Gln 0.4 diets relative to non Gln supplemented diets set to be 1.0. Bars indicate the 95% confidence interval (Fold change up - Fold change low). (*: *P* < 0.05)

**Fig. 3:**
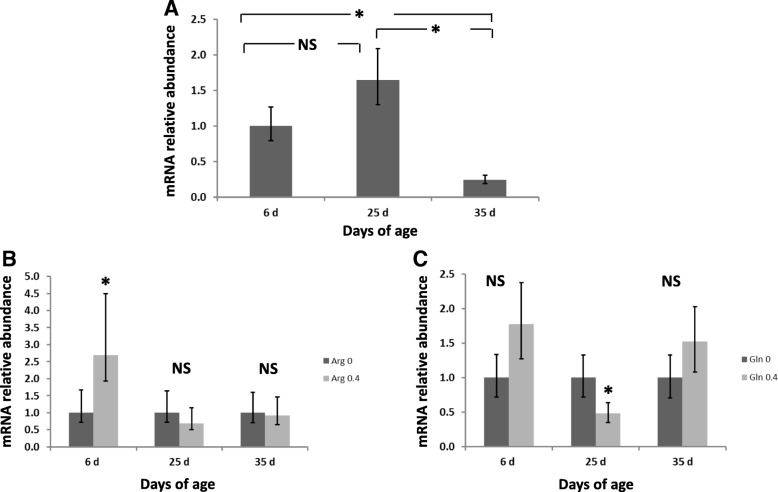
Interleukin (IL)-6 mRNA expression in intraepithelial lymphocytes isolated from rabbit Appendix. A significant effect of age (*P* < 0.001), and of the interactions Arg × Age (*P* = 0.016) and Gln × Age (*P* = 0.017) were found. **a** Relative gene expression values are fold change of 25 and 35 d relative to 6 d old rabbits set to be 1.0. **b** Relative gene expression values are fold change of rabbits fed Arg 0.4 diets relative to non Arg supplemented diets set to be 1.0. **c** Relative gene expression values are fold change of rabbits fed Gln 0.4 diets relative to non Gln supplemented diets set to be 1.0. Bars indicate the 95% confidence interval (Fold change up - Fold change low). (*: *P* < 0.05)

**Fig. 4:**
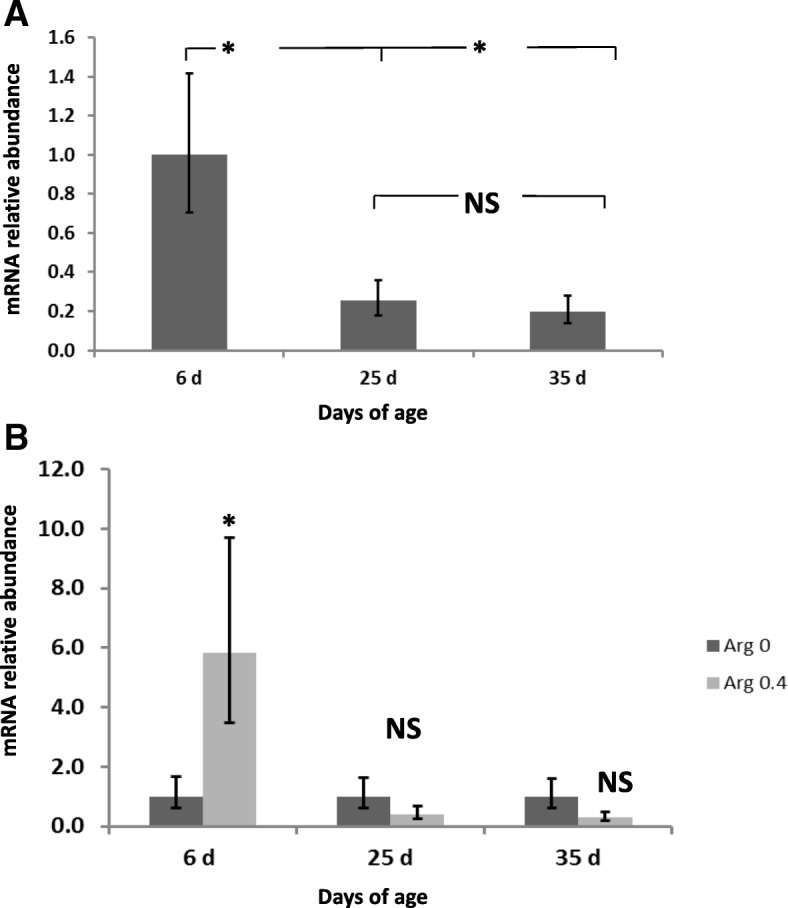
Interleukin (IL)-8 mRNA expression in intraepithelial lymphocytes isolated from rabbit Appendix. A significant effect of age (*P* < 0.001), and of the interaction Arg × Age (*P* < 0.001) were found. **a** Relative gene expression values are fold change of 25 and 35 d relative to 6 d old rabbits set to be 1.0. **b** Relative gene expression values are fold change of rabbits fed Gln 0.4 diets relative to non Gln supplemented diets set to be 1.0. Bars indicate the 95% confidence interval (Fold change up - Fold change low). (*: *P* < 0.05)

**Fig. 5:**
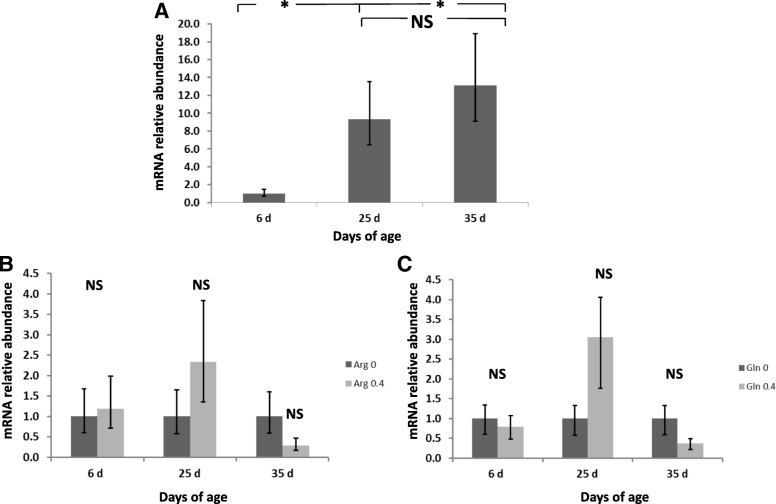
Interleukin (IL)-10 mRNA expression in intraepithelial lymphocytes isolated from rabbit Appendix. A significant effect of age (P < 0.001), and of the interactions Arg × Age (*P* = 0.027) and Gln × Age (*P* = 0.027) were found. **a** Relative gene expression values are fold change of 25 and 35 d relative to 6 d old rabbits set to be 1.0. **b** Relative gene expression values are fold change of rabbits fed Arg 0.4 diets relative to non Arg supplemented diets set to be 1.0. **c** Relative gene expression values are fold change of rabbits fed Gln 0.4 diets relative to non Gln supplemented diets set to be 1.0. Bars indicate the 95% confidence interval (Fold change up - Fold change low). (*: *P* < 0.05)

The expression of IL-2 in the appendix was significantly affected by age (*P* < 0.001) with an overexpression at 25 d compared to 6 and 35 d (Fig. 2). Moreover, a higher expression of IL-2 at 25 and 35 d of age was observed in rabbits fed the Gln diets (*P* = 0.017 for the interaction Gln × Age). The expression of IL-6 was significantly downregulated at 35d compared to 6 d and 25 d (*P* < 0.001) (Fig. 3). The expression of IL-6 at 6 d of age was higher in rabbits fed the Arg diets compared to the no supplemented diet (*P* = 0.016 for the interaction Arg × Age). However, a significant IL-6 downregulation was observed at 25 d of age in Gln fed animals compared to rabbits fed the no supplemented diets (*P* = 0.017 for the interaction Gln × Age). The expression of IL-8 in the appendix was affected by age (*P* < 0.001) with a significant downregulation at 25d and 35 d compared to 6 d of age (Fig. 4). Rabbits fed the Arg diets overexpressed IL-8 at 6 d of age (*P* < 0.001 for the interaction Arg × Age). Finally, the expression of IL-10 was significantly (*P* < 0.001) upregulated in rabbits at 25 and 35 d of age compared to those at 6 d (Fig. 5). An interaction Arg × Age and Gln × Age was found (*P* = 0.027) for IL-10 expression because of the upregulation observed at 25 d of age in rabbits fed the Arg and Gln supplemented diets compared to the no supplemented diet.

## Discussion

Individual dietary amino acids such as Gln and Arg are receiving much attention as functional nutrients for young animals as they promote gut health and development [3, 6]. It has been shown that dietary supplementation with Gln (0,5–5% w/w) at weaning modulates the piglet GALT function towards an immune-tolerant response increasing also the intestinal barrier performance [6]. Furthermore, dietary supplementation with Arg to gilts and piglets (10% and 0.4–0.8 w/w respectively) increased animal performance and immune function [24, 25]. We have previously reported a positive effect on litter size at birth and on litter weight at weaning in rabbit does supplemented with Arg or Gln but no effects on their kits weight at weaning [14]. The purpose of the present study was to assess if the exposure to Gln, Arg or their combination from pregnancy, through the maternal diet, to a post weaning supplemented diet, can stimulate litter performance, gut development and immune function. Feeding diets supplemented with 0.4% Gln, Arg or their combination to does and rabbits did not affect animal performance. Studies in piglets have shown that there is not always a productive enhancement with Gln supplementation despite the well reported positive effects of Gln on gut integrity and physiology [26]. In previous research, we were unable to detect significant differences on feed efficiency, daily gain or feed intake in young rabbits fed diets supplemented with 1% Gln [12]. However, studies with Arg seem to be more consistent regarding the positive effects of supplementing this amino acid on pre- and post-weaning pig performance [27]. A lack of effect of Arg on performance in our study compared to those in pigs might depend on species differences or dosage used. Moreover, as previously reported the combination of Gln and Arg showed no significant improvement on performance [12].

Intestinal bacterial translocation to MLN is a spontaneous event during rabbit development that peaks at around 6 days of age and then decreases as the gut barrier mechanisms mature [28]. In the present study, aerobic, facultative aerobic and anaerobic bacteria were isolated from MLN of rabbits at 6 d of age. Moreover, there was a tendency to reduce the total and facultative anaerobe translocation in the litters of does fed the Gln supplemented diets. Decreased bacterial translocation to MLN as the rabbit ages might be related to a higher degree of intestinal mucosal maturation and function triggered by the presence of milk components such as immune cells, Gln or epidermal growth factor [28]. Gut maturation includes an increased number of goblet cells, higher villus, or T lymphocyte proliferation [28, 29]. Sows fed diets supplemented with 2.5% Gln showed 265% higher concentration of this amino acid in the milk [30]. Therefore, it is plausible to relate the lower MLN bacterial counts in our study with a higher presence of Gln in the milk of does fed the Gln supplemented diets. However, we were unable to detect a higher number of goblet cells, villous height or T lymphocyte proportions in rabbits fed the Gln supplemented diets. Thus, future research with higher Gln supplementation is desired to corroborate the potential benefit of supplementing does with this amino acid on bacterial translocation.

The inclusion of Gln or Arg has been proven to support mucosal integrity by increasing or maintaining villous height and crypt depth in pre- and post-weaning healthy piglets [6]. In the present study we were unable to detect significant differences on gut histology and function with dietary supplementation of Gln, Arg or their combination as previously reported [12]. However, in agreement with studies in piglets [31] and rabbits [32, 33] a decrease in villous height was observed around weaning in the jejunum, although it may depend on the type of diet supplied [21]. Moreover, villous height in jejunum was maintained at pre-weaning levels in rabbits fed Gln. This has been previously described in pigs [31] consistent with the preventive role of Gln on villous atrophy around weaning. Crypt depth, the number of goblet cells and sucrose activity progressively increased with age as an indicative of gut maturity [32, 34], but were not affected by dietary means.

Glutamine plays a key role on lymphocyte function and cytokine production in healthy weaning piglets [6]. It is a source of energy for lymphocyte proliferation and modulates pro- and anti-inflammatory cytokine production in intestinal epithelial cells [35, 36]. Arginine also has an important role on immune cell function through the synthesis of NO favoring immune cell proliferation and activity [37]. The percentage of IEL CD45^+^CD4^+^ and CD45^+^CD8^+^ were not affected by dietary treatments, however their proportions tended to change with age. The proportions of CD4^+^ decreased from 6 to 25 days of age and then increased again. On the other hand CD8^+^ increased with age. This is in agreement with a decreased CD4^+^/CD8^+^ ratio in rabbit spleen, lymph nodes and circulation supported by a significant increase in CD8^+^ T-lymphocytes as the rabbit ages [38]. An increase in a controlled T cytotoxic response as the animal ages seems to be related to the normal function of the adaptive immune response [38]. Moreover, most of the IEL in the appendix are CD8^+^ regulatory T cells important to control inflammation and maintain immune tolerance [39]. The observed increase in CD8^+^ T lymphocytes with age is in agreement with a significant increase in IL-10 expression together with a down-regulation of the chemokyne IL-8. Both interleukins participate in the control of lymphocyte-mediated inflammatory responses, i.e. IL-10 attract CD8^+^ T lymphocytes and inhibits the CD4^+^ T lymphocyte migration by an inhibitory effect on IL-8 [40]. The commensal microflora in the appendix seems to play an important role in developing and maintaining a normal immune function by a direct interaction with lymphocyte function [39]. It was also noticeable the significant increase of IL-2 at weaning decreasing thereafter together with IL-6 expression. This cytokine expression pattern at weaning was also observed in mice, with a significant increase of IL-2, IL-6 and IL-10 in small intestine lamina propria total lymphocytes after weaning [41]. The exposure to food antigens together with changes on gut microbiota results in a transient Th1 vs. Th2 balanced cytokine profile that helps the GALT mature and differentiates [41]. Dietary Gln fed to 25d old rabbits upregulated IL-2 and IL-10 while downregulated IL-6 expression. The concentration of extracellular Gln has been shown to increase IL-2 production and signaling in T lymphocytes [42]. Also, in IL-1b-stimulated duodenal biopsies the addition of Gln significantly increased IL-10 expression and reduced that of IL-6 and IL-8 [43]. Given the important role of IL-2 in regulatory T cell homeostasis and function [44] it is plausive that the cytokine profile observed in our study is the result of a more moderate and balanced Th1-Th2 response at weaning in rabbits fed the Gln supplemented diets. In contrast, Arg was shown to be more reactive at 6 days of age displaying a pro-inflammatory profile with increasing IL-8 and IL-6 expression in the appendix. In early weaned piglets, a positive effect of dietary Arg was observed by enhancing cellular and humoral immunity [25]. They observed lower IL-8 serum concentrations but higher IL-8 gene expression in the piglet spleen with no significant changes on IL-6 concentration or expression. Arginine derived NO might enhance the immune response by means of increase lymphocyte proliferation, and macrophage and natural killer activity [37]. However, NO overproduction can also produce intestinal mucosa injury and dysfunction [37]. Moreover, NO production seems to be of relevance to control intestinal epithelial injury and restitution in neonatal necrotizing enterocolitis [45].

## Conclusions

In conclusion, despite a lack of effect on performance and mortality the inclusion of 0.4% Gln has a positive effect by maintaining intestinal villous height and modulating the cytokine profile in the Appendix at weaning. The supplementation with Arg or Arg + Gln at the selected doses in this study did not exert positive effects on rabbit intestinal health.

## Data Availability

The datasets used and/or analysed during the current study are available from the corresponding author on reasonable request.

## Abbreviations

Arg: Arginine
Gln: Glutamine
IEL: Intraepithelial lymphocytes
MLN: Mesenteric lymphoid nodes
